# Handheld computers for self-administered sensitive data collection: A comparative study in Peru

**DOI:** 10.1186/1472-6947-8-11

**Published:** 2008-03-19

**Authors:** Antonio Bernabe-Ortiz, Walter H Curioso, Marco A Gonzales, Wilfredo Evangelista, Jesus M Castagnetto, Cesar P Carcamo, James P Hughes, Patricia J Garcia, Geoffrey P Garnett, King K Holmes

**Affiliations:** 1 School of Public Health and Administration, Universidad Peruana Cayetano Heredia, Lima, Peru; 2 Division of Biomedical and Health Informatics. School of Medicine, University of Washington, Seattle, USA; 3 School of Science. Universidad Peruana Cayetano Heredia, Lima, Peru; 4 Department of Infectious Disease Epidemiology, Imperial College London, London W21PG, UK; 5 Departments of Epidemiology and Medicine and Center for AIDS and STD, University of Washington, Seattle, USA

## Abstract

**Background:**

Low-cost handheld computers (PDA) potentially represent an efficient tool for collecting sensitive data in surveys. The goal of this study is to evaluate the quality of sexual behavior data collected with handheld computers in comparison with paper-based questionnaires.

**Methods:**

A PDA-based program for data collection was developed using Open-Source tools. In two cross-sectional studies, we compared data concerning sexual behavior collected with paper forms to data collected with PDA-based forms in Ancon (Lima).

**Results:**

The first study enrolled 200 participants (18–29 years). General agreement between data collected with paper format and handheld computers was 86%. Categorical variables agreement was between 70.5% and 98.5% (Kappa: 0.43–0.86) while numeric variables agreement was between 57.1% and 79.8% (Spearman: 0.76–0.95). Agreement and correlation were higher in those who had completed at least high school than those with less education. The second study enrolled 198 participants. Rates of responses to sensitive questions were similar between both kinds of questionnaires. However, the number of inconsistencies (p = 0.0001) and missing values (p = 0.001) were significantly higher in paper questionnaires.

**Conclusion:**

This study showed the value of the use of handheld computers for collecting sensitive data, since a high level of agreement between paper and PDA responses was reached. In addition, a lower number of inconsistencies and missing values were found with the PDA-based system. This study has demonstrated that it is feasible to develop a low-cost application for handheld computers, and that PDAs are feasible alternatives for collecting field data in a developing country.

## Background

In the last 20 years, different methodologies have appeared to improve data collection quality in sensitive topics [1]. Sexual behavior is largely determined by social, cultural, religious, moral, and legal norms and constraints [2]. In addition, a complete evaluation of sexual behavior includes knowledge, attitudes, risk behaviors and more, all of which are very difficult to evaluate because individuals tend to deny involvement in socially undesirable behaviors to avoid stigmatization [3]. Social desirability or self-presentation interviewer can affect reports about sexual behaviors as well as other sensitive behaviors. This might change the analysis for non-responses items [4].

Systematic reviews of research in sexual behavior have been published recently. Most publications note that the validity and reliability of data collected by computers depend on variables like age group of participants and the types of sensitive questions [5]. Many studies have been designed to develop methods to maximize the accuracy of reporting risky sexual behaviors for sexually transmitted diseases (STD) and HIV infection in the general population [6]. Although most of these studies have included pen-and-paper self-completed interviews, about 20 years ago, computer-assisted interviewing (CAI) and computer-assisted self-interviewing (CASI) appeared as an alternative to paper questionnaires for the collection of reliable information on sensitive behaviors [7-9]. Some types of CASI include audio, video, or telephone enhancements [10]. These have been used to assess general risk [11], patient history [12], and a variety of health related data [13-15].

Particularly in developing countries, data collection methods are needed that are reliable, inexpensive, and do not require extensive technological expertise [16]. Applicability of portable computers for surveys in the general population could be limited due to the cost of computers, software costs, and the risk of data loss due to mishandling, malfunction or theft. In spite of these difficulties, handheld CASI is emerging as a new tool for collection of risk-behavior data due to its advantages, including portability and energy efficiency [17], reduction on interviewer bias, real time authentication and validity, conditional branching, and minimization of data transcription and transfer errors [18].

The objective of this study is to present two experiences with the use of Personal Digital Assistants (PDA) in CAI and CASI for the collection of sex-related sensitive data from participants of a household based survey, and to compare these data to similar data collected in paper questionnaires.

## Methods

### Study design and setting

Two cross sectional surveys were undertaken in Ancon, a district of Lima, Peru (August 2005 and August 2006). In both surveys, a sample of clusters was selected; then a census of each household in the selected clusters was conducted. Within each household, eligible individuals (male or female, 18–29 years, literate, and in the household at the moment of the interview) were selected. Participants provided verbal informed consent prior to participate, and completed a detailed questionnaire on sexual practices. Participation in both surveys was anonymous.

### Definitions

Low educational level was defined as having had no more than a secondary school education. A low income was defined as having a personal monthly income less than or equal to 140 dollars.

### Questionnaire characteristics and interview

The questionnaire explored past and current STD symptoms and signs, as well as sexual practices. Topics were approximately 110 closed-ended questions and were filled-in by the participant confidentially.

In the first cross-sectional survey, each participant completed the questionnaire in two formats: paper and PDA. Participants were first asked to complete the paper-based self-applied questionnaire, and then to fold it and put it into a locked voting bag. Then they received a short training session (approximately 2–3 minutes) on the use of the PDA, and completed a PDA-based questionnaire [11]. In the second cross-sectional survey, field workers were assigned to teams of two alphabetically based on their last name. Within each team, the first interviewer conducted the interview with the electronic format while the second interviewer conducted the interview with the paper format. As a result, half of participants answered the PDA questionnaire and the other half responded the paper-based questionnaire.

### Program used in handheld computers (PDA-PREVEN)

The PDA software program was built using Open-Source tools and contained the same sequence of questions as the paper format. The GNU Compiler Collection (GCC), a General Public License Free Software application, was used for building Palm OS applications in C and C++ using the cross-compiler libraries and SDK that can be downloaded at the Palmsource website (ACCESS Linux Platform) [19]. The questionnaire structure was built from a Comma-separated value (CSV) file, used by a small application (written in the C++ language) running under the Debian Linux Operating System, to produce a Palm executable application using the aforementioned cross-compiler. Low-cost Palm Zire-31^® ^PDAs were used and data and applications were transferred to them using Palm's HotSync program.

The questionnaire contained a set of data entry types (pop-up lists, multi-option answers, one-option answers, etc.). Participants entered data using those types of entry options. They chose answers from a list previously established. Participants did not have to entry text using the pen stylus. Some questions were only asked if the response to a previous question met a predefined rule. Participants were required to select a response prior to moving to the next question. The program also allowed participants to return to previous questions within the same section to modify their answers.

**Figure 1:**
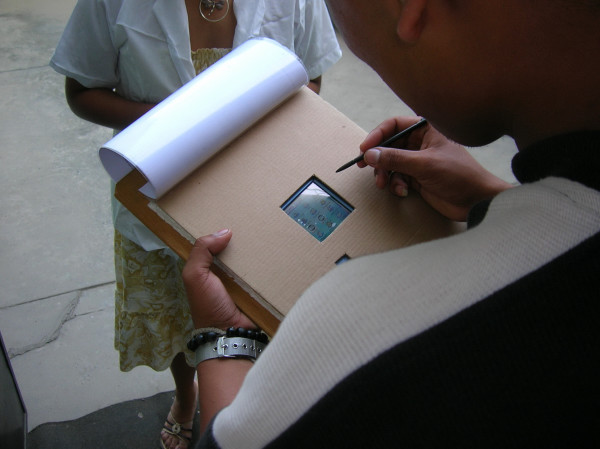
**An example a clipboard with PDA**. Photograph shows the form of interviewing through a PDA put into a clipboard.

During fieldwork, each handheld computer was inserted into a wooden and Styrofoam clipboard to shield it from possible damage and to conceal it (Figure 1).

### Data management and statistical analysis

All paper questionnaires from both surveys were double entered into a Microsoft Access 2000 template (Microsoft Corporation, Washington, USA), while PDA data was transferred to a computer through a HotSync operation (synchronization), converted into a CSV format using a program based on C, and then reorganized into a single database within Microsoft Visual FoxPro 7.0 (Microsoft Corporation, Washington, USA). Statistical analysis was performed in STATA 8.0 for Windows (STATA Corporation, Texas, USA). A subset of questions from the questionnaire was selected based on their sensitivity for comparisons between the methodologies.

For the first survey, categorical variables were compared using Kappa coefficient analysis while numeric variables were compared using Spearman Rho correlation. Overall agreement (for both categorical and numeric measures) was defined as the number of equivalent responses in both questionnaires divided by the total number of responses. Also, the correlation of variables according to sex and education level of participants was calculated.

For the second survey, the same categorical variables were compared using χ^2 ^test or Fisher's exact test, while numeric variables were compared using Student's t test. In this case, we also compared the number of missing values, the number of inconsistent responses and the duration of the interview. A missing value was defined as the lack of response, while an inconsistent response was defined as a discordant answer between two related questions. The duration of the interview was evaluated as the time measured between the beginning and the ending of the self-applied questionnaire.

## Results

### Study participants

The first survey enrolled 200 participants. Ten pairs of questionnaires (5%) could not be matched because of miscoding, and therefore, 190 self-applied paper and PDA questionnaires were analyzed. Ninety four (49.5%) of the participants were male and the mean sample age was 22.9 (SD: 3.4).

The second survey enrolled 198 participants. Similarly, a total of 98 records were recovered from PDA, while 100 records were attained by the paper format. Ninety nine (50.0%) of the participants were male and the mean age sample was 22.7 (SD: 3.4). Population characteristics of both survey groups are shown in Table 1.

**Table 1:** Population characteristics of both survey groups

**Variables**	**First survey 2005 N (%)**	**Second survey 2006 N (%)**
Age (years)		
Mean ± SD	22.9 ± 3.4	22.7 ± 3.4
Sex		
Male	94 (49.5%)	99 (50.0%)
Educational level		
Low (≤ high school)	140 (73.7%)	146 (73.7%)
Marital status		
Single	117 (61.6%)	138 (69.7%)
Married/Cohabiting with partner	68 (35.8%)	60 (30.3%)
Separated/Divorced	5 (2.6%)	0 (0.0%)
Monthly income		
Low income	151 (80.3%)	159 (83.7%)
Previously interviewed		
Yes	--	10 (5.3%)

### Evaluation of responses in the first survey

The comparison of the responses to the two formats is shown in Table 2. General agreement between paper and PDA self-applied questionnaires was 86%. Agreement for categorical variables ranged from 70.5% to 98.5%, with Kappa coefficients from 0.43 to 0.86. For numerical variables, agreement varied from 57.1% to 79.8%, with a Spearman's Rho coefficient between 0.76 and 0.95 depending on the question evaluated. Likewise, the comparison between paper and PDA self-applied questionnaires according to sex of participants only demonstrated slight differences between men and women. However, participants with higher education level consistently had better agreement in both categorical and numerical variables than those with less education (Table 3).

**Table 2:** General correlation between responses of PDA and paper self applied questionnaires (first survey)

**Nominal or categorical questions:**	**N**	**Agreement**	**K coefficient**	**P**
Last 12 months, secretion from penis/bad smelling vaginal discharge	173	84.2%	0.54	< 0.0001
Last 12 months, sores or ulcers in penis or genitals	180	93.7%	0.43	< 0.0001
Have you ever had sexual intercourse	177	87.9%	0.67	< 0.0001
Pregnancy ended in an abortion ^a^	63	95.2%	0.81	< 0.0001
Spontaneous abortions in the first three months of pregnancy ^a^	63	95.2%	0.76	< 0.0001
Have you ever had sexual relation with a prostitute ^b^	95	94.4%	0.86	< 0.0001
Have you ever had sexual relation with a transvestite or man ^b^	95	86.3%	0.48	< 0.0001
Last sexual partner, male or female ^b^	73	98.5%	0.79	< 0.0001
Last sexual partner, how long know this person before you had first sex	129	70.5%	0.63	< 0.0001

**Numeric questions:**	**N**	**Agreement**	**Spearman R**	**P**

Age of first sexual intercourse	129	79.8%	0.95	< 0.0001
Number of different people you have had sex in your life	122	79.1%	0.93	< 0.0001
Last 3 months, how many times you had sex with your last partner	31	67.7%	0.76	< 0.0001
Last 3 months, how many times you did not use condoms with your last partner	7	57.1%	0.81	< 0.05

^a ^Question only applied for women.

^b ^Question only applied for men.

**Table 3:** Correlation between responses according to educational level of participants (first survey)

	**≥ High school**		**< High school**	
**Nominal or categorical variables:**	**Agreement**	**Kappa**	**Agreement**	**Kappa**
Last 12 months, secretion from penis/bad smelling vaginal discharge	97.1%	0.89	83.1%	0.44
Last 12 months, sores or ulcers en penis or genitals	100.0%	1.00	97.4%	0.65
Have you ever had sexual intercourse	96.1%	0.90	92.0%	0.58
Pregnancy ended in an abortion ^a^	100.0%	1.00	96.8%	0.78
Spontaneous abortions in the first three months of pregnancy ^a^	100.0%	1.00	96.8%	0.78
Have you ever had sexual relation with a prostitute ^b^	100.0%	1.00	96.8%	0.78
Have you ever had sexual relation with a transvestite or man ^b^	92.5%	0.55	78.8%	0.42
Last sexual partner, male or female ^b^	97.5%	0.66	100.0%	1.00
Last sexual partner, how long know this person before you had first sex	89.6%	0.68	91.1%	0.79

	**≥ High school**		**< High school**	
**Numerical variables:**	**N**	**Spearman**	**N**	**Spearman**

Age of first sexual intercourse	65	0.96	54	0.93
Number of different people you have had sex in your life	64	0.91	51	0.98
Last 3 months, how many times you had sex with your last partner	17	0.95	14	0.58
Last 3 months, how many times you did not use condoms with your last partner	-	-	-	-

^a ^Question only applied for women.

^b ^Question only applied for men.

### Evaluation of responses in the second survey

Table 4 shows the comparison of responses for the second survey using the same questions evaluated in the first one. It is important to notice that two questions evaluated in this survey ("have you ever had sex with a female sex worker" and "age of first sexual intercourse") had p-values near 0.05. When the number of inconsistencies was evaluated, the mean in the paper format was 1.93 (SD: 1.98), while it was 0.08 (SD: 0.54) in the PDA format (p < 0.0001). Similarly, the mean number of missing values was 0.85 (SD: 1.35) in the paper questionnaire and 0.29 (SD: 1.02) in the PDA format (p = 0.001). Finally, the average time in answering in the paper format was 9.68 (SD: 12.98) minutes, whilst in the PDA format was 7.20 (SD: 9.38) minutes (p = 0.065). However, in spite of rapidness, 6.9% of interviews had to reset the electronic device during the field work.

**Table 4:** Response rates comparison between PDA and paper self applied questionnaires (second survey)

**Nominal or categorical questions:**	**PDA self-applied questionnaire**	**Paper self-applied questionnaire**	**p**
Last 12 months, secretion from penis/bad smelling vaginal discharge (yes)	12 (13.6%)	11 (12.0%)	0.83
Last 12 months, sores or ulcers in penis or genitals (yes)	4 (4.2%)	4 (4.4%)	1.00
Have you ever had sexual intercourse (yes)	77 (81.1%)	66 (72.5%)	0.22
Pregnancy ended in an abortion (yes) ^a^	8 (22.9%)	9 (24.3%)	1.00
Spontaneous abortions in the first three months of pregnancy (yes) ^a^	6 (17.1%)	9 (24.3%)	0.57
Have you ever had sexual relation with a prostitute (yes) ^b^	8 (19.1%)	16 (36.4%)	0.09
Have you ever had sexual relation with a transvestite or man (yes) ^b^	2 (4.8%)	2 (4.7%)	1.00
Last sexual partner (male) ^b^	0 (0.0%)	1 (2.4%)	0.72
Last sexual partner, how long know this person before you had first sex (< 30 days)	20 (26.3%)	21 (28.4%)	0.98

**Numeric questions:**			

Age of first sexual intercourse	17.81 ± 3.43	16.89 ± 2.55	0.07
Number of different people you have had sex in your life	3.17 ± 3.57	3.27 ± 2.92	0.85
Last 3 months, how many times you had sex with your last partner	4.75 ± 6.18	2.94 ± 4.52	0.16
Last 3 months, how many times you did not use condoms with your last partner	3.72 ± 5.62	2.92 ± 2.84	0.63

^a ^Question only applied for women.

^b ^Question only applied for men.

## Discussion

The results of the first survey show an overall kappa coefficient of 0.86 suggesting an almost perfect agreement between PDA and paper responses [20]. This finding supports the utility of PDA-PREVEN for collecting survey data in the field. The correlation was greater for numerical than for nominal variables. In addition, observed agreement for numeric variables had less concordance when the overall number of responses was smaller. Other studies aimed at young populations have found similar results, perhaps due to the willingness of young people to use new technological devices such as computers, PDAs, cell phones, etc [2,3]. Since young Peruvian people are not familiar with the use of handheld computers, rather than desktops and Internet, we decided to conduct a short training session before collecting data. In addition, we conducted the training to recognize the type of possible models of questions and responses, and to avoid PDA screen damage by pressure. Likewise, the high agreement could be explained by the use of a set of questions with a pre-defined menu of alternatives as a part of the program. Besides, the agreement in those who had completed at least high school was higher than those who did not, which could be in accordance with the skill level required to operate electronic devices and the ability to respond to both questionnaires in a consistent manner.

In the second survey, data collected by both techniques were very similar, which is supported by the fact that the statistical analysis found no significant difference between groups. Although the responses to the two aforementioned questions were near to the usual significance level, those were not considered significant after their alpha level was corrected by the Bonferroni's procedure (cut-off for 15 comparisons: 0.003) [21,22]. When comparisons were performed to evaluate data accuracy through the number of missing values and inconsistent answers, these were statistically lower in the PDA group. Similar to previous studies, responding the questionnaire in PDA format was about 25% faster than paper format [18,23,24]. However, this difference was not statistically significant. Overall, the PDA avoids inconsistencies during data collection, helps preserve data integrity, and performs at least as well as the paper questionnaire.

In previous studies [25,26], technical malfunction has been described as the main disadvantage with the use of PDA format. In this study, 6.9% of interviews had to reset the electronic device during the field work. We designed our PDA application to have an option to return to the question where the interview was interrupted, which minimized data loss.

In general, our results agree with studies using PC-based CASI or audio-CASI for collecting data from general population [2,27], blood donors [28,29], and for surveys on alcohol or drug consumption [11,30]. In a previous study using PDAs conducted by Fletcher [11], agreement attained between both kinds of questionnaires was higher (about 96%). However, the information was collected twice by trained staff members, whereas in our surveys both questionnaires were self-applied and answered by the participants after a short training period. For this study, all questions were closed-ended, which could help explain the high level of correlation. At the same time, our design reflects the actual setting and experience of conducting a field survey.

The major strength of this study is the application of a PDA software program using Open Source tools for collecting data, and two different methodologies to evaluate it, which allows us to develop a low-cost system, tailored more closely to our needs and specifications without the limitations of proprietary systems. To our knowledge, this is the first report that evaluates the usefulness of using a software program built with Open Source tools in a PDA to collect data about sexual behavior in the field in Peru. The first methodology allows us to demonstrate an almost perfect correlation between the two sorts of questionnaires since the same questions were applied twice to the participants, reducing the inter-observant variation. The second methodology allows us to compare the rate of responses, the rate of consistencies, the rate of missing values, and the duration between both sorts of questionnaires, which were not evaluated in the first survey.

Most of the studies with PDAs have used commercial and expensive programs to create data entry forms [1,11]. The use of programs based on Open Source tools has been previously described in rural areas [31] to allow paramedical health workers to view large databases. Using these tools, other authors have developed databases and web-applications for collecting, storing, and querying biological pathway data [32] or managing information in biomedical studies [33,34]. In our case, we needed an application for collecting information rather than simply viewing it. Notably, during fieldwork we did not lose any PDA, probably due to the ability to conceal them within the clipboard.

Our study has several limitations. One of the most important is that inconsistencies between both questionnaires may be due to non-selective misclassification because of recall problems. Difficulties in remembering information during the interview might have been present even if the participants would have asked to fill out paper-based surveys twice or handheld computer surveys twice. Unfortunately, this issue was not evaluated in the surveys. Later studies should be performed to assess if less recall problems are present using handheld computers versus paper-based questionnaires. Also, some bias could have been introduced in the first survey because all the participants were asked to complete the paper-based before PDA questionnaire. However, we believe that whether the half of participants had firstly responded to the PDA questionnaire, they would not have paid attention to the paper questionnaire or would have left without answers due to the boredom caused by answering the questions twice, which would have been more unfavorable to the paper questionnaire. Another limitation was the small sample size, which did not allow us to compare some questions between groups. Although we found some differences related to education level, agreement and correlation were high in low and high educational level groups.

## Conclusion

Handheld computers were useful for collecting information about sexual behavior in young people in Peru. The two surveys administered have demonstrated that it is feasible to develop a low-cost application for handheld computers to collect sexual behavior data. Our study suggests that PDAs are feasible alternatives to paper forms for field data collection in a developing country.

## Competing interests

The author(s) declare that they have no competing interests.

## Authors' contributions

AB, WHC and MAG conceived the idea. AB and MAG drafted the paper. MAG analyzed the results. CPC contributed his expertise in epidemiological studies and participated in the design of the study. JMC and WE contributed their expertise in Open Source technology and PDA use. JPH contributed his expertise in statistical analysis. PJG, GPG, and KKH are senior authors who conceived the overall idea and guided the progress of this manuscript. All the authors read and approved the final manuscript.

## Pre-publication history

The pre-publication history for this paper can be accessed here:


